# PROSPECTIVE ASSOCIATIONS BETWEEN MULTIDIMENSIONAL NEIGHBORHOOD CONTEXTS AND PSYCHOSOCIAL OUTCOMES IN LATER LIFE

**DOI:** 10.1093/geroni/igae098.2958

**Published:** 2024-12-31

**Authors:** Ethan Siu Leung Cheung, Ming Wen, David Curtis, Sara Grineski, Yehua Dennis Wei

**Affiliations:** University of Utah, Salt Lake City, Utah, United States; University of Hong Kong, Pok Fu Lam, Hong Kong; University of Utah, Salt Lake City, Utah, United States; University of Utah, Salt Lake City, Utah, United States; University of Utah, Salt Lake City, Utah, United States

## Abstract

Despite extensive literature having reported on contextual place effects on older adults’ individual health and well-being outcomes, the longitudinal relationships between multidimensional neighborhood environments and diverse psychosocial outcomes remain less known. This study addresses two questions: 1) What are the prospective associations between multidimensional neighborhood environments and psychosocial outcomes (i.e., purpose in life, depression, generalized anxiety disorder, life satisfaction) among older adults? 2) Do these relationships differ based on age groups (Youngest-old 65-74, Middle-old 75-84, Oldest-old 85 or over) and biological sex? This investigation adopts individual data from waves 2 (2004-2006) and 3 (2013-2014) of the core sample in Midlife Development in the United States, supplemented with an oversample of African Americans in Milwaukee. Tract-level neighborhood data come from multiple public sources and are measured in 2010. The analytic sample includes 1,249 individuals aged 65 and above at wave 3. Time-random-effect linear and logistic regressions are used to examine the main place effects, and interaction analyses by age and sex are conducted for each psychosocial outcome. Results suggested that area-level economic disadvantage is associated with a lower level of purpose in life. Social disorganization is a significant risk factor for generalized anxiety disorder. Some of these effects significantly vary by female. The findings highlight the importance of neighborhood social, built, and natural environments for various psychosocial outcomes and underscore the need for age-sensitive environmental interventions and programs to enhance psychosocial well-being in later life.

